# The history and typification of *Liliumhumboldtii* J.H.Krelage (Liliaceae)

**DOI:** 10.3897/phytokeys.182.70099

**Published:** 2021-09-14

**Authors:** James Compton, Mark W. Skinner

**Affiliations:** 1 Spilsbury Farm, Tisbury Row, Salisbury SP3 6RU, UK Spilsbury Farm Salisbury United Kingdom; 2 Skinner and Associates, 1275 SE River Forest Rd., Milwaukie, OR 97267 USA Skinner and Associates Milwaukie United States of America

**Keywords:** Duchartre, Krelage, Leichtlin, *Liliumhumboldtii*, morphological key, nomenclature, Regel, Roezl, typification

## Abstract

The history of the Californian *Liliumhumboldtii* J.H.Krelage, its initial discovery and confusion in literature over its collection, distribution and naming are discussed. Neotypes are designated for the names *Liliumhumboldtii* and *Liliumbloomerianum* Kellogg. Lectotypes are designated for the names Liliumcanadensevar.puberulum Torr. and L.bloomerianumvar.ocellatum Kellogg.

## Introduction

The Californian lily, *Liliumhumboldtii*, was first recognised to be a new and distinct taxon by the botanist John Torrey when he received material in New York sent to him from California in 1854 by the botanist John Milton Bigelow. Bigelow was employed on the government-sponsored 35^th^ Parallel Pacific Railroad Survey of the plants found along one of the proposed Pacific Railroad routes across the USA, led by Lieutenant Amiel Weeks Whipple. Torrey published the name Liliumcanadensevar.puberulum Torr. in the belief that the lily was a variety of *L.canadense* L., a species native to the eastern USA (Torrey 1856: 146). Torrey added, however, “If the characters given above prove constant, this fine lily must be considered distinct from *L.canadense*” (Torrey 1856: 147).

The lily was identified as a new species sometime before 1860 by Dr Albert Kellogg (1813–1887) one of the seven founding members of the Californian Academy of Sciences. Kellogg had seen this species in cultivation in the garden of Hiram Green Bloomer (1819–1874) a botanist and another of the founders of that prestigious Academy. Kellogg was so impressed with the lily that he had it painted (possibly by Bloomer who was known to be an accomplished artist) and exhibited it in the halls of the Academy in 1860 where he proposed the name *L.bloomerianum* for it, but he did not validly publish that name for another 12 years (Kellogg 1872: 160).

This paper looks at the history of this species and follows the course of its rediscovery by a Czech plant collector in California, its arrival in Germany and its distribution to The Netherlands, France, England and Russia. We address some of the misinformation and confusion that has surrounded this magnificent Californian lily since its first recorded discovery.

### Benedikt Roezl (1824–1885), the collector

The Czech traveller, botanist and plantsman, Benedikt Roezl is known largely for the immense quantity of orchids that he collected in Mexico and in various countries of South America. He started his horticultural career in 1836 in the garden of Tetschen [Děčín] Castle, the home of Leopold Graf von Thun und Hohenstein in Bohemia (Czech Republic), then later worked for Louis Van Houtte in Gentbrugge (Belgium) in 1846 where he became the new chef de culture of Van Houtte’s école d’horticulture de Gand (Ghent).

Roezl collected in North America on his extended route across the USA in 1869 while travelling to California from New York, via St Louis, Chicago, Omaha, Cheyenne and Truckee (Mabberley 1985: 452). Roezl wrote a report of his Californian journey, including the discovery of *L.humboldtii* and sent it to Eduard Ortgies, Superintendent of the University of Zurich Botanic Garden and Roezl’s de facto agent, who published the account in “Gartenflora” (Ortgies 1871: 108). In it, Roezl wrote (translated from the German):

“A commercial gardener in San Francisco told me about a very beautiful lily with yellow, red-dotted flowers which occurs near Nevada City. I hired two Chinese to help me and having made the acquaintance of Dr. Tiling, a doctor who is interested in the local flora, within eight days I had many bulbs of this lily. If I am permitted, I should like to give this lily the name *L.humboldtii* as it was found on the 100^th^ birthday of von Humboldt. This beautiful lily has golden-yellow flowers with red and black spots, leaves clustered in whorls and stems five to six feet tall and bears up to 35 flowers” (Ortgies 1871: 108).

Ortgies added a footnote stating that Herr. Leichtlin handled the purchase and import of the lily whose flowers were noticeably long-lived (Ortgies 1871: 108). The Dr. Tiling mentioned by Roezl must have been the physician and botanist Heinrich Sylvester Theodor Tiling (1818–1871).

Roezl added additional information on his discovery in a short autobiography of his expeditions and collections for the “Gardeners’ Chronicle” published on 18 July 1874 (Roezl 1874). In that year, he returned to Europe to settle in Smichow, Bohemia [Smíchov, a district of Prague]. He wrote about his arrival in the USA only four years after the end of the American Civil War and having lost one of his arms while demonstrating a new sugar-cutting machine in Cuba in 1868:

“Afterwards I proceeded to New York to start on my Californian travels over the Rocky Mountains and the Sierra Nevada. I discovered here the new lilies *L.washingtonianum*, *puberulum*, *parvum* and *humboldtii*; the latter I found on the hundredth memorial day of Alexander von Humboldt and hence named one of the species after him. The lily in question does not come from the Humboldt County as some catalogues assert” (Roezl 1874: 73).

Roezl’s discovery of this new species would therefore have been on 6 May 1869 although he never published a validating description of his new lily.

### Jacob Heinrich Krelage (1824–1901), the author of the name

In the world of horticulture, the great beauty and stature of this Californian lily was very quickly recognised. Roezl’s collection crossed the Atlantic to Europe in the same year that it had been collected. It was first validly named *Liliumhumboldtii* in Haarlem, The Netherlands, in 1870, some five and a half thousand miles from its native habitat in the Sierra Nevada. Jacob Heinrich Krelage, son of the nurseryman Ernst Heinrich Krelage (1786–1855), was a keen and very successful commercial grower of bulbous plants. He placed two advertisements in the “Gardeners’ Chronicle and Agricultural Gazette” on 22 and 29 October 1870 for his *E.H.Krelage en zoon* nursery listing *Liliumhumboldtii* as a new and interesting *Lilium*. His description in both cases was short: “from California, 5 feet high, golden flowers spotted with purple, £1 each” (J.H.Krelage 1870: 1402; 1435). There is, however, just enough descriptive matter to satisfy Art. 38.1 of the International Code of Nomenclature (ICN; Turland et al. 2018). Art. 38.1 is “one of the most difficult provisions of the ICN to apply” (J. Wiersema, pers. comm., 24 May 2021), thus the authors of this note affirmed with a number of experts involved in the current ICN, including Dr John Wiersema (US) and Dr John McNeill (E), the sufficiency of J.H.Krelage’s brief description which predates that of the English nurseryman William Bull of King’s Road, Chelsea in London. Bull, whether aware or not of Krelage’s acquisition, provided an equally short description of this recently imported Californian lily less than three months later, on 14 January 1871 (see Typification below).

**Figure 1.**
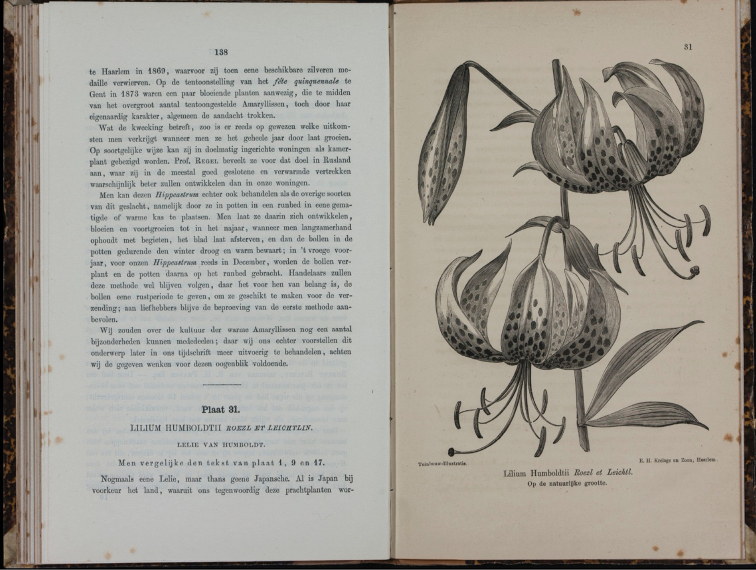
Liliumhumboldtiisubsp.humboldtii in “De Tuinbouw-Illustratie Tijdschrift voor Tuinbouw en Plantkunde” plate 31. (Krelage 1874).

Jacob Krelage waited another four years before writing a full account of *L.humboldtii* in his “De Tuinbouw-Illustratie Tijdschrift voor Tuinbouw en Plantkunde” (“The Illustrated-Horticulture Magazine for Horticulture and Botany”). He also included a plate (see Fig. 1) to accompany the comprehensive descriptive text and provided much valuable information about its history (J.H.Krelage 1874: 138, t. 31). Of further interest, the description, published by J.H.Krelage in 1870, is attributed to *E.H.Krelage en zoon*, but without specifying any individual of the business as the author of the name. Therefore, we have invoked external evidence (ICN Art. 46.9) to establish that J.H.Krelage was the only extant member of the Krelage firm (E.H.Krelage having died in 1855) and is, therefore, the author of *Liliumhumboldtii*.

### Pierre Duchartre (1811–1894), lily writer and taxonomist

The French botanist, Pierre Etienne Simon Duchartre, was one of the founders of the Société Botanique de France in 1854. From a young age he had been fascinated by the morphology of the genus *Lilium* and, in particular, its bulb structure. He wrote extensively on the species that were cultivated in the garden of the bulb connoisseur Max Leichtlin in Karlsruhe. His articles, each entitled “Observations sur le Genre Lis”, appeared in nine separate instalments in the French “Journal de la Société Impériale et Centrale d’Horticulture de France” Ser. 2 in vols. 4 and 5 (Duchartre 1870; Duchartre 1871). In the seventh of these instalments, he wrote (translated from the French):

“*Liliumhumboldtii* is one of the most beautiful discoveries made by Benedikt Roezl and one of the most precious introductions into the garden of Maximilien Leichtlin” (Duchartre 1871: 94).

Duchartre added that on 14 May 1870 Leichtlin had written a letter to him informing him that B. Roezl had found the plant at Devil’s Gate, in a ravine that ran alongside the Pacific Railroad next to a river with many rapids, before arriving at Wintah Station from where the train goes on to Mormon City. This misleading information, imparted second hand from Roezl via Leichtlin, has been the source of much confusion and is discussed below under the heading The Utah Mystery.

Duchartre also added that the description he is providing was based on two young and rather meagre specimens which he owed to the kindness of his great friend M. Leichtlin and explained that his friend Leichtlin had witnessed the species come into flower for the first time in the month of July 1870 (Duchartre 1871: 95). Finally, Duchartre provided a comprehensive Latin description and diagnosis in the firm belief that, judging from his earlier correspondence with Leichtlin, the plant known as “*Liliumhumboldtii* Roezl et Leichtlin in litt.” had not yet been formally described. He added after the description:

“Hab. in Californiae montibus Sierra Nevada dictis, ubi a cl. Roezl detectum est ab eo cum cl. et amicissimo Max Leichtlin communicatum (v.v.c.)” [It grows in the Sierra Nevada mountains of California, where it was found by the famous Roezl and it was communicated to me by him along with my great and famous friend Max Leichtlin. v.v.c.] (v.v.c = *vidi vivam cultam*: I have seen it alive in cultivation) (Duchartre 1871: 97).

**Figure 2.**
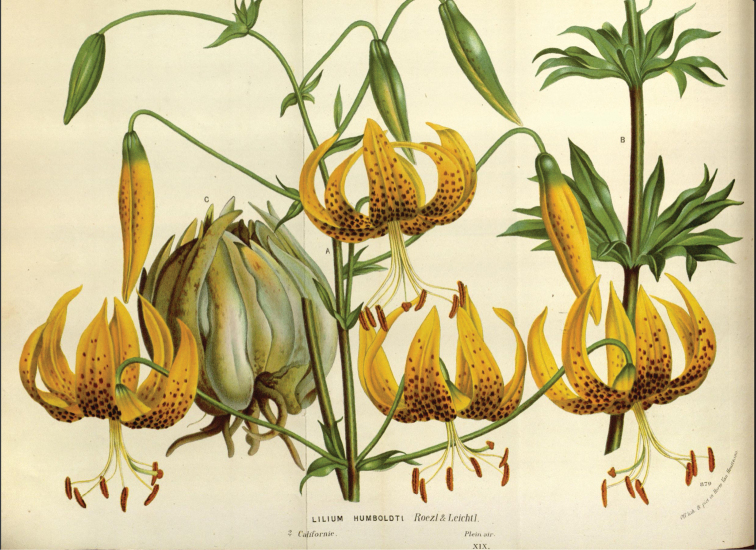
Illustration of Liliumhumboldtiisubsp.humboldtii, painted from Louis Van Houtte’s nursery garden and published in “Flore des Serres” 19: t. 879. (Duchartre 1873b).

Duchartre had maintained a long correspondence with Max Leichtlin (see below) in Karlsruhe [formerly Carlsruhe], also with J. H. Krelage in Haarlem and with the great nurseryman Louis Van Houtte in Gentbrugge (Duchartre 1873a: 2). Two years after his informative paper on *L.humboldtii* (Duchartre 1871), he repeated the salient extracts from that paper in a separate article that he wrote for Van Houtte’s journal “Flore des Serres”. A coloured plate (see Fig. 2) of a plant that had been cultivated in Van Houtte’s garden was included (Duchartre 1873b t. 879).

Amongst Duchartre’s correspondence, now kept in the Lenhardt Library of the Chicago Botanic Garden, is a handwritten note on *L.humboldtii* including a sketch by him of the bulb, which showed Duchartre’s particular interest in bulb diversity within the genus. The note simply says (translated from the French):

“On the 19 February 1870, I received by the post from Mr. Max Leichtlin of Carlsruhe, a beautiful lily bulb with the following letter: “*I am sending you today a bulb of the rare, new L.humboldtii of this magnificent genus. The bulbs came to me from the Sierra Nevada of North America. You can see at first glance the singular growth next to the developing bulb which sinks into the soil each year. It has orange flowers spotted with purple and carries sometimes as many as 30 flowers of a quite remarkable beauty. P.S. my plant only provisionally carries the name L.humboldtii.*”

The illustration of the bulb drawn by Duchartre in pencil also bears a short note by him (translated from the French):

“Liliumhumboldtii (provisional name) sent from Carlsruhe by M. Max Leichtlin on 19 February 1870. The bulb is as I received it. The bulb is situated on the side of a stem fragment from under which emerge several now dried out roots. It has the general form of a martagon with large scales, thinner, almost flat, whitish, rather speckled towards the top”.

### Maximilien Leichtlin (1831–1910), bulb grower extraordinaire

Max Leichtlin was one of the greatest growers of rare bulbs during the 19^th^ century. He was the third son of Eduard Leichtlin, founder in 1823 of a successful paper-making business with his brother in Karlsruhe. Max Leichtlin briefly joined the family business before beginning his lifelong career in horticulture, first as an apprentice in the gardens of the Schloss in Karlsruhe for the Grand Duke of Baden, then he went to the Königliche Gartenakademie [Royal Garden Academy] in Potsdam. In 1850, he travelled around Europe and South America to enhance his botanical knowledge, returning to Europe in 1856 (Hooker 1883: 1; Nelmes and Cuthbertson 1931: 223). He worked for two years in the nursery of Louis Van Houtte in Gentbrugge. On the death of his eldest brother, Leichtlin rejoined the family paper business until leaving the business again in the 1860s in order to lay out his own garden near to the Stadtgarten Karlsruhe [now Zoologischer Stadtgarten] where he cultivated many rare plants. His garden included some 250 different lilies, many acquired from his ever-burgeoning list of cosmopolitan contacts, Roezl being one of them. Leichtlin also distributed plants amongst his correspondents including to Duchartre in France and Eduard Regel (see below) in Russia.

Leichtlin had to leave Karlsruhe in 1873 owing to development in the southern part of the city. He moved to Baden-Baden 40 km south of Karlsruhe where he acquired a new garden near the Neuen Schlosses and continued to grow his rare and unusual species. His reputation by this time was huge and he was given honorary fellowship of the Royal Horticultural Society in London as well as being awarded the Veitch Memorial Medal by the Society. Leichtlin produced from this garden a series of sale catalogues of the plants that he grew there. It is worth noting that, in his first list of *Lilium*, he credited his friend Duchartre with the authorship of the name “*L.humboldtii* Duchartre” (Leichtlin 1874: 16), but later, in his undated list of “Lilien”, he attributed the name to Roezl as “*L.humboldtii* Roezl” without any description or additional information in either case (Leichtlin suppl. 1: 2, pre–1873).

### Eduard Regel (1815–1892), the St Petersburg connection

Eduard August von Regel was a prolific German horticulturist and botanist who worked during his early career in botanic gardens in Göttingen, Bonn and Berlin. In 1842, he was appointed head of the botanic garden in Zürich. In 1852, he founded and edited the magazine “Gartenflora”, in which he described several new lily species. In 1855, he became a research botanist at the Imperial Botanic Garden in St Petersburg and, from 1875, he was its director and remained so until his death.

**Figure 3.**
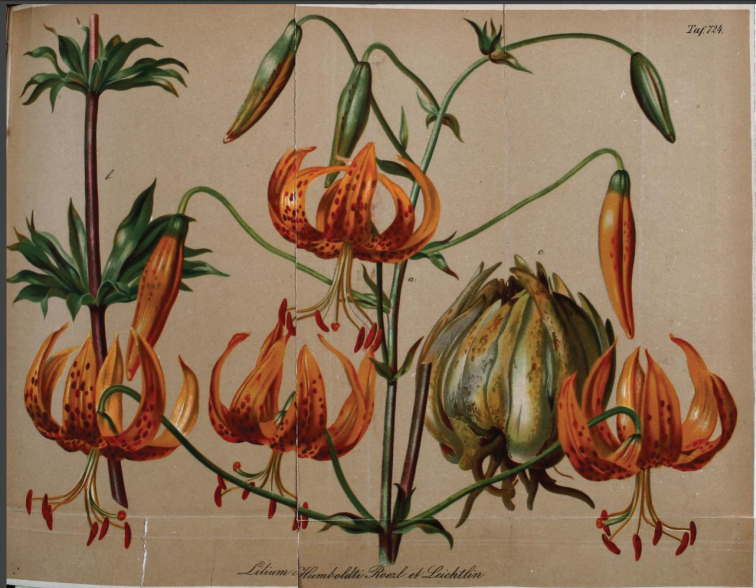
The neotype of *Liliumhumboldtii* from Louis Van Houtte’s nursery garden, published in “Gartenflora” 21: t. 724 (Regel 1872).

In vol. 21 of his “Gartenflora”, he described *Liliumhumboldtii* with a fine coloured illustration (Regel 1872: 161–163, t. 724, see Fig. 3). This must have been sent to him courtesy of Louis van Houtte as the same image was reproduced a year later for Duchartre (Duchartre 1873b t. 879, see typification below). Regel more or less repeats the text previously published by Duchartre (Duchartre 1871: 94–97), but adds some important information regarding the source of his own plants of the species from which the plate was prepared. His text included (translated from the German):

“We owe the depiction of the beautiful lily that our table presents here to our honoured friend Leichtlin. The bulb shown at c in the table is based on a living specimen in the garden. B. Roezl discovered this excellent and beautiful lily in California in the Sierra Nevada at Devil’s Gate, a wild mountain valley through which a river with many rapids falls, along which the Pacific Railway runs through the mountains. Mr M. Leichtlin bought from Mr Roezl all of the bulbs which he had collected, with the exception of a few specimens, which were obtained through the intermediary of Mr M. Leichtlin for the Imperial Botanic Garden in St. Petersburg”.

Regel’s comments on the provenance of the species once again have added to the confusion surrounding its origin. It seems that he is describing the Devil’s Gate in Utah, but mistakenly placing it in the Sierra Nevada in California. This error probably occurred from misinformation supplied to him by Leichtlin (see The Utah Mystery below). In addition, it is not clear in which garden the living bulb shown at c was in cultivation (but see discussion on the illustrations in Typification below).

## The Utah mystery

The geographic range of Liliumhumboldtiisubsp.humboldtii and Liliumhumboldtiisubsp.ocellatum is now well established as being restricted to the Sierra Nevada, coastal ranges and Channel Islands of California (Abrams 1923; Skinner 2002). How is it, therefore, that two of Roezl’s lily collections were described by Eduard Regel (Regel 1870: 321) and as mentioned above by Duchartre (via Leichtlin) as coming from the Mormon State i. e. Utah?

The first of Roezl’s collections, originally named *Liliumroezli* Regel, but now recognised to be *L.pardalinum*, was, according to Regel:

“Die beistehen Lilie ward von Roezl im Felsengeberg in der nähe des Mormonen-Staates entdeckt und is mit *L.superbum* L. zunächst verwandt.” [The lily shown here was discovered by Roezl in the Rocky Mountains near the Mormon State and was originally thought to be related to *L.superbum* L.” (Regel 1870: 321).

The second collection, already discussed, of *L.humboldtii* includes the statement by Leichtlin that “Roezl found it at Devil’s Gate, in a ravine that ran alongside the Pacific railroad next to a river with many rapids, before arriving at Wintah Station from where the train goes on to Mormon City”. The construction of the Union Pacific Railroad linking the east with western USA in 1869 took place from two directions. The line running eastwards from San Francisco in California ran through the Sierra Nevada range, across the State of Nevada and across the north of Utah. The westwards line from Wyoming ran through the Wahsatch [Wasatch] mountains along the Weber Canyon and Devil’s Gate Pass to Uintah Station, then on to Ogden and finally up to Promontory Junction. The opening ceremony for the joining of these two lines at Promontory was on 9 May 1869. Roezl himself stated that he had collected several lilies, including *L.humboldtii*, in the Sierra Nevada, but he clearly also travelled along the route of the Union Pacific Railroad through Devil’s Gate Pass which is east of the mining town of Uintah (Leichtlin’s Wintah) only three days before the joining up of the two lines. His journey down the course of the Weber River towards the Great Salt Lake must have been by horse or on foot as the line was not yet up and running.

Another curious anomaly in the two statements is that no species of the genus *Lilium* from western USA has been found as far east as the State of Utah; indeed, it is the only State in the continental US with no native *Lilium*. The localities of *Liliumhumboldtii* are on the western slopes of the Sierra Nevada and along the coastal ranges in southern California some 600 miles (ca. 950 km) west of Devil’s Gate in Utah. The distribution of *L.pardalinum* Kellogg is in California and southernmost Oregon. According to Roezl’s report to Ortgies on his Californian travels (described above), we now know that the lily was actually collected in the Sierra Nevada in Nevada County near Nevada City (Ortgies 1871: 108).

There are, however, two species of *Fritillaria* that do occur in the Wasatch Mountains that span the States of Utah and Wyoming through which the Devil’s Gate Pass runs: *F.atropurpurea* Nutt. and *F.pudica* (Pursh) Spreng. It would seem likely that, through the various communications between Roezl, Leichtlin and his associates, the provenance of the lilies and the fritillaries may have been mistakenly mixed up. In one of his undated catalogues of the plants grown in his garden, Max Leichtlin mentioned under the heading Knollen und Zwiebel-Gewächse [Tubers and Bulbs] the entry: “*Fritillaria* species from Devil’s Gate” (Leichtlin undated Catalogue pre–1873: 8).

To add an extra layer of confusion, there is also a Devil’s Gate in Mono County, California, but it is outside the range of both *L.pardalinum* and *L.humboldtii* and has nothing to do directly with our history.

## Typification

No original material was cited in the nurseryman Jacob Krelage’s catalogue advertisement and it is extremely unlikely that a specimen would have been collected. The publication, however, must serve as the protologue for the name *L.humboldtii* (J.H.Krelage 1870: 1402). To recapitulate from above, the description, albeit very short, is enough to validate the name (Art. 38.1, Turland et al. 2018; J. Wiersema, pers. comm., 24 May 2021) and predates the short advert also placed in the “Gardeners’ Chronicle” the following year by another bulb enthusiast and nurseryman William Bull of London on 14 January 1871. Bull’s description under the heading ‘New Lilies’ might have been one of the nursery entries that had later prompted Roezl to correct the misrepresentation of the locality of the species:

“*Liliumhumboldtii* – a splendid species from Humboldt County, California, growing about five feet high and producing large golden-yellow flowers, which are spotted with purple. Good bulbs. Price 1 guinea each” (Bull 1871: 35).

Krelage’s brief description also predates Roezl’s provisional name suggested in his own report published by his agent Ortgies in “Gartenflora” (Ortgies 1871: 108), as well as the comprehensive description provided by Duchartre later that year (Duchartre 1871: 97).

Three illustrations exist of plants grown directly from Roezl’s collections. The history of the two published coloured images, resulting directly from Roezl’s collection in California, is rather confusing.

Upon his receipt of the bulbs from Roezl, Leichtlin must have forwarded some bulbs very soon after their arrival in Karlsruhe to Louis Van Houtte in Belgium. This information is revealed by comparing Regel’s illustration (Fig. 3) in “Gartenflora” (Regel 1872: t. 724) with the illustration that was published of this species in “Flore des Serres” a year later (Fig. 2) and which is clearly a reproduction of the same image (Duchartre 1873b: t. 879). The later illustration in “Flore des Serres” actually includes the statement:

“*L.humboldtii* Roezl et Leichtl. – Regel Gartenflora, juin et août 1872, ubi tabulam nostram reperimus” [Regel Gartenflora, June and August 1872, where our plate was to be found].

This plate was initially prepared from bulbs grown in Van Houtte’s garden in Gentbrugge as revealed by the legend “Off. lith. & pict. in Horto Van Houtteano” shown bottom right. In effect, Regel predated Duchartre to the publication of the same image. The two images differ from each other in the format of the lettering which was altered from capitals and with the addition of “Californie” and “plein air” [out in the open] in “Flore des Serres” (Duchartre 1873b: t. 879) to small and italicised in “Gartenflora” (Regel 1872: t. 724). The shading in the latter is also darker.

A few of Leichtlin’s bulbs had also been forwarded to Regel in St. Petersburg where they were cultivated in the Imperial Botanic Garden. From these bulbs, two herbarium specimens were prepared. These can be seen in LE: LE-01072601 and LE-01072602.

The third illustration appeared another year later (Fig. 1) in “De Tuinbouw-Illustratie”, the journal for the nursery of *E.H.Krelage en zoon*. Uncoloured, this was prepared from the first bulbs cultivated in Haarlem, The Netherlands that had been sent to Jacob Krelage from Leichtlin (Krelage 1874: 138–142, t. 31).

Although these illustrations are historically linked with Roezl’s original collection of this species, none can be considered as original material and, therefore, a neotype must be chosen that can be representative of the species (J.McNeill, pers. comm., 7 April 2021). The illustration accompanying Regel’s text in “Gartenflora” (1872: t. 724) is, therefore, chosen here as the type for the name (see Fig. 3).

## Taxonomic conspectus

### Lilium humboldtii

Taxon classificationPlantaeLilialesLiliaceae

J.H.Krelage, Gard. Chron. 1870: 1402 (1870)

6BCA7AD5-E09B-5494-B6D6-1B00FCC3111B

#### Neotype.

Designated here: [Icon], Gartenflora 21: t. 724 (1872).

### Key to subspecies of *L.humboldtii*

**Table:** 

1	Bulbs off-white, occasionally flecked with purple, scales always unsegmented; sepals and petals orange, spots magenta, without aureolated lighter red margins; foothills of Sierra Nevada	**Liliumhumboldtiisubsp.humboldtii**
2	Bulbs often purplish, scales segmented; sepals and petals yellow or light orange, spots red or magenta, aureolated with lighter red margins; southern California	**Liliumhumboldtiisubsp.ocellatum**

### Lilium humboldtii subsp. humboldtii

Taxon classificationPlantaeLilialesLiliaceae

3BEA363D-9000-5FED-A188-F91478308F53

≡ Lilium canadense var. humboldtii (J.H.Krelage) Baker, Gard. Chron. 1871: 1165 (1871)
= Lilium canadense var. puberulum Torr., Pacific Railr. Rep. 4 pt.5 no.4: 146 (1856) **Lectotype** designated here: USA, **California**, “*K.T.Hartweg* 2004” (GH, lecto!) [GH-00106407]; syntypes: USA, **California** “border of meadows, Antelope Creek, one of the tributaries of the Upper Sacramento, 23 May 1846 *Col. Frémont* 490” (NY, syn!) [NY-0008523]; USA, **California** “near Butte Creek in the Sacramento Valley, 16 June 1848, *Hartweg 2004*” (K, syn.! × 2).
≡ Lilium puberulum (Torr.) Duchartre, J. Soc. Centr. Hort. France ser. 2, Vol. 4: 217 (1870).

#### Diagnostic description.

**Bulb**: off-white, occasionally flecked with purple; scales unsegmented; stem roots absent. **Racemes**: 1–33(–40) flowered. **Flowers**: with sepals and petals orange, speckled with magenta, spots distributed distally or more proximally; pollen rust, rust-brown, rust-orange, occasionally to warm tan, becoming yellowish. **Seeds**: 114–225 per capsule.

#### Distribution.

U.S.A., California (Amador, Butte, Calaveras, El Dorado, Fresno, Mariposa, Nevada, Placer, Tehama, Tuolumne, Yuba). Liliumhumboldtiisubsp.humboldtii is distributed from Tehama County south to Calaveras County; reports from further south are erroneous.

#### Ecology.

Flowering summer (mid-June to early August), frequently in forest openings of Ponderosa pine forest (*Pinusponderosa* Douglas ex C.Lawson) and chaparral openings; (200–1100 m).

#### Illustration.

“Gartenflora” 21: t. 724 (1872) see Fig. 3. https://www.biodiversitylibrary.org/item/125746#page/215/mode/1up

### Lilium humboldtii subsp. ocellatum

Taxon classificationPlantaeLilialesLiliaceae

(Kellogg) Thorne, Aliso 9: 195. 1978

F73CC982-86F0-574E-B211-C20DC2EE98D8

**Figure 4.**
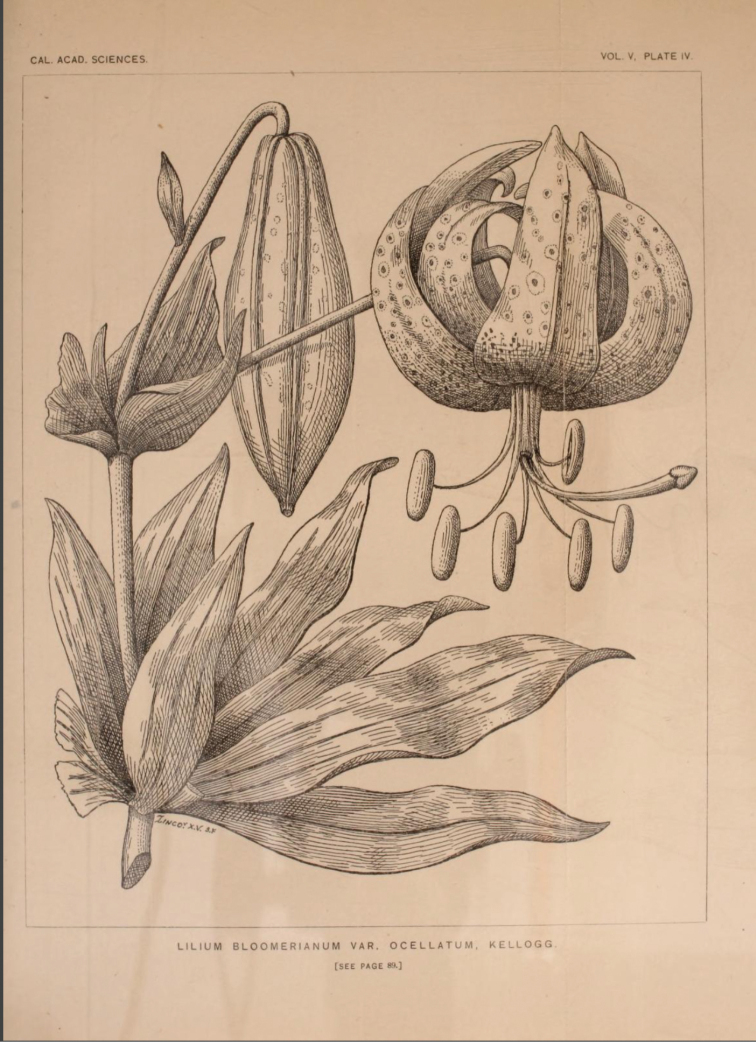
Illustration chosen as lectotype for the name Liliumbloomerianumvar.ocellatum Kellogg, in Proceedings of the California Academy of Sciences 5: t. 4 (Kellogg 1873).

Basionym: Lilium bloomerianum var. ocellatum Kellogg, Proc. Calif. Acad. Sci. 5: 88 (1873). **Lectotype** designated here: [Icon] Proc. Calif. Acad. Sci. 5: 88 (1873: t. 4, see Fig. 4).
≡ Lilium humboldtii var. ocellatum (Kellogg) Baker, Journ. Linn. Soc. (Botany) 14(76): 245 (1874).
≡ Lilium ocellatum (Kellogg) Beane, Contr. Dudley Herb. 4: 358 (1955).
= Lilium bloomerianum Kellogg, Proc. Calif. Acad. Sci. 4: 160 (1872). Type: USA, **California**, [Icon] not seen, probably destroyed; **Neotype** designated here: USA, California, *A.Kellogg & W.G.W.Harford 978* coll. 1868–1869. (US neo!) [US-03945856].
≡ Lilium humboldtii var. bloomerianum (Kellogg) Purdy, J. Roy. Hort. Soc. 26: 354 (1901).
= Lilium humboldtii var. magnificum Purdy, J. Roy. Hort. Soc. 26: 353 (1901). Type not seen
= Lilium fairchildii M.E.Jones, Contr. W. Bot. 16: 39, 26 (1930) **holotype**: USA, **California**, San Diego, Mt. Palomar, west of Hot Springs, 13 July 1929, *M.E.Jones* 24762 (RSA, holo!) [RSA-0000358]

#### Note 1.

In the absence of any type material, the description of *L.bloomerianum* by Kellogg of the bulbs as purplish and his statement “This is the most magnificent lily of the Pacific coast” indicate that he was describing L.humboldtiisubsp.ocellatum. This is reinforced by Purdy’s description of L.humboldtiivar.bloomerianum (with *L.bloomerianum* Kellogg also cited) as occurring in San Diego County which is within the range of subsp. ocellatum, but outside that of subsp. humboldtii. The only material indicated in the protologue as type of the name *L.bloomerianum* is the illustration donated by an unknown donor to the California Academy of Sciences which may have perished in the earthquake and fire of 1906 (Emily Magnaghi; Seth Cotterell, pers. comm.). A neotype has, therefore, been chosen.

It could be argued that, under Art. 36.1 (Turland et al. 2018), the name *L.bloomerianum* is not a validly published name as it could be seen as a “provisional name” that has been merely proposed in anticipation of its future acceptance. In his report to the California Academy of Science, Kellogg stated “Out of respect to its time-honored cultivator, Mr. H. G. Bloomer, he offered the provisional name of *Liliumbloomerianum*”. In this case, however, Kellogg added the comment “This lily is the most magnificent lily of the Pacific Coast” and the diagnostic sentence “This lily is easily discriminated from all others in any stage of its growth”. These comments are more than merely provisional. Moreover, the formal heading of “On *Liliumbloomerianum*”, as well as the full description, indicates that Kellogg is validly describing the species.

#### Note 2.

Original herbarium material of Liliumbloomerianumvar.ocellatum is stated in the protologue to have been gathered by *William George Willoughby Harford* of the U. S. Coast Survey from Santa Rosa Island. No specimen has been located. It is possible that it might also have perished in the earthquake and fire at CAS in 1906 (Emily Magnaghi, pers. comm.). We have, therefore, chosen the illustration (plate 4) that accompanies the text, as the lectotype of the name (Kellogg 1873 t. 4, see Fig. 4).

#### Diagnostic description.

**Bulb**: often purplish, especially apically; scales notched, segmented with 2–5 poorly defined segments or occasionally unsegmented; stem roots frequently present. **Racemes**: 1–25-flowered. **Flowers**: with sepals and petals yellow or light orange, speckled with large red or magenta spots aureolated with light red margins, spots larger and their margins wider and lighter towards the apex; pollen tan or peach, becoming yellow or tan-yellow, occasionally tan-orangish or rust. **Seeds**: 150–252 per capsule.

#### Distribution.

U.S.A., California (Anacapa Island, Santa Cruz Island, Santa Rosa Island, Los Angeles, Orange, Riverside, Santa Barbara, San Bernardino, Ventura). In addition to the mainland (see counties above), it occurs on the larger northern Channel Islands, where it is the only native lily.

#### Ecology.

Flowering late spring–summer (mid-May to July). Oak canyons, chaparral; 0–1800 m elev. Liliumhumboldtiisubsp.ocellatumis similar tosubsp.humboldtii, but the yellowish sepals and petals with widely margined spots, lighter-coloured pollen and purplish bulb with notched scales are distinctive.

#### Illustration.

**Figure 5.**
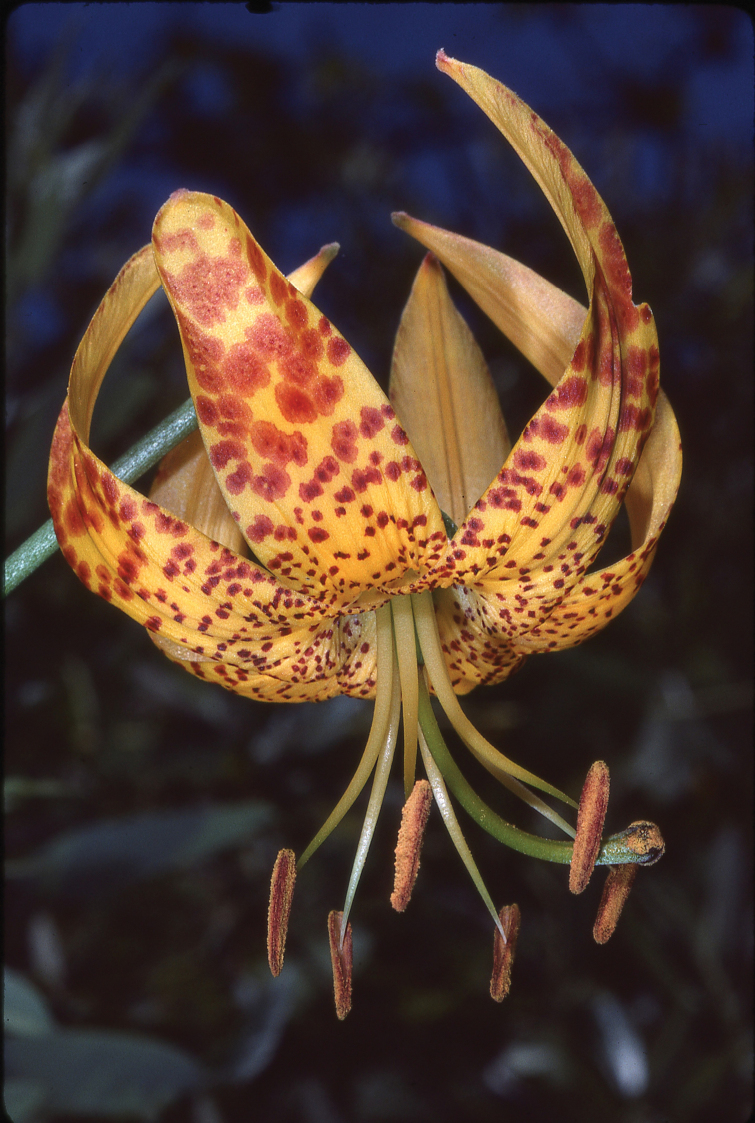
Representative specimen of Liliumhumboldtiisubsp.ocellatum in Peutz Valley near San Diego, California, showing the ocellated markings on the floral segments of the subspecies. (Mark Skinner).

Liliumhumboldtiisubsp.ocellatum Photo. Mark Skinner (Fig. 5): Peutz Valley, east of San Diego, California

## Epilogue

It took a European nurseryman to provide a formal name for a beautiful Californian lily species for the first time. It is worth noting that, despite its almost immediate appeal in Europe, there are very few records of this lily being in cultivation in its native country within the first decades of its rediscovery and distribution by Roezl. This paucity may have been due to the instability of the western part of the USA after the Mexico-American War, which was not finalised until 1848. This volatility was followed just over a decade later by the immense disruption caused by the American Civil War from 1861 to 1865. Roezl’s journey, therefore, took place only a few years after the dust had settled from the Civil War and during that period of American history when the west of the vast country was finally being conveniently connected by the railroad to the east.

One early American record of the cultivation of *L.humboldtii* is that of the nurseryman L. B. Case of Richmond, Indiana where *L.humboldtii* was listed as having flowers “yellow, with dark spots. 75c each, $7 per doz.” (Case 1877: 23). This was, therefore, eight years after Roezl had collected and distributed his plants and six years after its first description in a Dutch nursery catalogue.

## Unpublished material

The Duchartre Collection of manuscript notes, drawings and photographs on lilies (1870–1880), Chicago Botanic Garden Lenhardt Library, Illinois State Library digital archives http://www.idaillinois.org/digital/collection/ncbglib01/id/24727

## Supplementary Material

XML Treatment for Lilium humboldtii

XML Treatment for Lilium humboldtii subsp. humboldtii

XML Treatment for Lilium humboldtii subsp. ocellatum
